# The population structure of the *Cryptosporidium parvum* population in Scotland: A complex picture

**DOI:** 10.1016/j.meegid.2007.10.010

**Published:** 2008-03

**Authors:** Liam J. Morrison, Marianne E. Mallon, Huw V. Smith, Annette MacLeod, Lihua Xiao, Andy Tait

**Affiliations:** a Wellcome Centre for Molecular Parasitology, University of Glasgow, Glasgow Biomedical Research Centre, 120 University Place, Glasgow G12 8TA, UK; b Scottish Parasite Diagnostic Laboratory, Stobhill Hospital, Glasgow, UK; c Division of Parasitic Diseases, National Centre for Infectious Diseases, Centres for Disease Control and Prevention, GA, USA

**Keywords:** *Cryptosporidium parvum*, Population genetics, Microsatellite repeats, Minisatellite repeats, Genotype

## Abstract

We genotyped 297 Scottish *C. parvum* samples using micro- and minisatellites. Treated as a single population, the population structure was epidemic. When regional populations were analysed, there was evidence of sub-population structure variations. This was dependent upon excluding sub-groups exhibiting significant genetic distance from the main population, implying genetic sub-structuring. We tested the hypothesis that these sub-groups originated outside the UK and demonstrated that one sub-group clustered with Peruvian samples. A geographically comprehensive panel of isolates would fully confirm this result. These data indicate limited sub-structuring within a small geographical area, but substantial sub-structuring over larger geographical distances. Host movement influences parasite diversity and population structure, evidenced by strong correlation (*r*^2^ = 0.9686) between cattle movements and parasite diversity. Thus, the population structure of *C. parvum* is complex, with sub-populations differing in structure and being influenced by host movements, including the introduction of novel multilocus genotypes from geographically distinct regions.

## 1 Introduction

*Cryptosporidium parvum* is a pathogen of humans, causing acute gastroenteritis that is usually self-limiting, but which can be life-threatening for immunodeficient individuals (Hunter and Nichols, 2002). It is also of veterinary importance, particularly in neonatal animals (de Graaf et al., 1999). Transmission is via water, food, and both person-to-person and zoonotic faecal/oral routes. Traditionally viewed as a promiscuous infectious agent, with human infections arising from the parasite population in domestic animals, *C. parvum* has recently been split into several species. Species identification has revealed a number of relatively host-specific *Cryptosporidium* genotypes, with 16 accepted species (Xiao et al., 2004), although the predominant species infecting humans are *C. parvum* and *Cryptospoidium hominis*. The current study examines *C. parvum sensu stricto*, which infects humans and other mammals.

The population structure of several parasites has been examined using micro- and minisatellite markers (Anderson et al., 2000; MacLeod et al., 2000). Indeed, within the Apicomplexa, parasites have strikingly different population structures. A clonal example, with infrequent genetic recombination resulting in linkage disequilibrium between genotypically different strains, is *Toxoplasma gondii* (Howe and Sibley, 1995). *Theileria parva* illustrates epidemicity, whereby random mating, unrestricted genetic exchange and linkage equilibrium is masked by the expansion of genetically identical parasites (Oura et al., 2005). *Plasmodium falciparum* in regions of high transmission exhibits frequent genetic exchange, linkage equilibrium and a state of panmixia (Anderson et al., 2000). However, the latter study illustrates the complexity of sub-structuring, with some sub-populations being epidemic and others panmictic, apparently dependent upon the transmission intensity.

Population genetic studies involving *Cryptosporidium* have only recently been undertaken. Intraspecific polymorphism of micro- and minisatellite markers has been identified for *C. parvum* and *C. hominis* (Aiello et al., 1999; Caccio et al., 2000, 2001; Mallon et al., 2003a,b; Tanriverdi et al., 2006). The population structure of *C. parvum* was considered clonal (Awad-El-Kariem, 1999), until human and bovine *C. parvum* samples from a region of Scotland were examined (Mallon et al., 2003a) using micro- and minisatellite analysis. The parasites isolated from cattle represented a randomly mating or panmictic population, providing the first evidence for genetic exchange in this parasite. The situation in *C. parvum* isolated from humans was different, in that although the most common multilocus genotypes (MLG) were present in the cattle samples, analysis of these MLGs in human outbreaks showed an epidemic population structure. However, a subset of human *C. parvum* MLGs was genetically distinct and not found in cattle, indicating either cycling of these particular MLGs within the human population, or an unidentified source of infection. The analysis was expanded with 242 additional samples (Mallon et al., 2003b) from two further geographical areas in Scotland and, although there was no evidence for geographical or temporal sub-structuring, the study confirmed that the *C. parvum* human population was epidemic, and the *C. parvum* bovine population panmictic. The genetically distinct subset of *C. parvum* isolates identified in humans in Aberdeenshire was also present in Dumfriesshire, and again was not present in livestock-derived samples.

We have analysed the same *C. parvum* samples, using an expanded panel of markers. When allele frequencies of the seven markers used in the previous studies are examined, four markers have one allele represented in most samples, with a few distinct alleles at low frequency. This near monomorphy within markers leads to the possibility of a type 2 error, because with one predominant allele the frequency of allele combinations between pairs of loci predicted by random genetic exchange would not be significantly different from that if mating was not occurring. To robustly test for panmixia, we developed further polymorphic microsatellite markers to investigate the previous conclusions. The additional markers allow a more robust examination of geographic sub-structuring and the role of genetic exchange in these populations. Analysis was extended to a geographically distinct subset of *C. parvum* isolates to determine potential sub-structuring over larger geographical distances.

## 2 Materials and methods

### 2.1 Parasite isolates

The samples analysed were those used in the previous studies by Mallon et al. (2003a,b), prepared as purified oocyst lysates (Nichols and Smith, 2004). Of the 347 *Cryptosporidium* species samples used in the previous studies (180 in Aberdeenshire, 72 in Orkney, 97 in Thurso, and 69 in Dumfries and Galloway), 297 were included in the present study. The *C. hominis* samples from Aberdeenshire analysed previously (Mallon et al., 2003a) were not included. For several *C. parvum* samples insufficient material remained to allow amplification of the 3 new microsatellite loci, and therefore as a result the final number of *C. parvum* samples used in our data set was 70 from Aberdeenshire, 68 from Orkney, 96 from Thurso and 63 from Dumfries and Galloway. These samples were isolated from humans, cattle and sheep. The origin of the samples was known to the level of the postcode. All of the samples analysed were *C. parvum* as determined previously (Mallon et al., 2003b). A set of more geographically diverse samples was also analysed, consisting of 12 Peruvian *C. parvum* samples from human infections. All samples were *C. parvum* as defined by RFLP analysis with the COWP gene (Spano et al., 1997).

### 2.2 PCR primer and conditions

Seven of the markers, and associated PCR conditions, were those used previously; ML1 (Caccio et al., 2001), GP15 (Strong et al., 2000), MS1 (Khramtsov et al., 1995), TP14, MS9, MS5 and MS12 (Mallon et al., 2003b). Three further microsatellite markers were developed; MM5, MM18 and MM19. Respective primer pairs were MM5A (GGAGAAGATAAGCTAGCCGAATCT) and MM5B (CCTGGACTTGGATTTGGACTTACACC), MM18A (CTTTCTGGAGGGTTTGTTCCTCC) and MM18B (CTTCCTGATGATCCAGGCCAAGC), and MM19A (GATTCTGTCAACTTTGAATTCAG) and MM19B (CCAACCCCGAATTCATTTCCAAC). PCR reactions were performed under the previously described conditions (Mallon et al., 2003a,b). The template was an optimised dilution of purified oocyst lysate. Reactions were performed in a Robocycler 96 (Stratagene). Cycling conditions were 95 °C for 50 s, 50 °C for 50 s, and 65 °C for 60 s, for 30 cycles. PCR products were resolved by electrophoresis of 3% Nusieve^®^ GTG (Cambrex) agarose gels, and visualised by staining with 0.2 μg/ml ethidium bromide under UV illumination.

### 2.3 Allele identification and multilocus genotype classification

The size of each PCR product was determined by separation on a capillary-based sequencer (ABI 3100 Genetic Analyser; Applied Biosystems). The inclusion of size standards (GS500; Applied Biosystems) allowed sizing of alleles by Genescan^®^ software. Each allele was assigned a number and an MLG designated by the combination of alleles at each locus. If a sample contained multiple MLGs, i.e. was a mixed infection, the predominant allele for each marker was scored, based on peak height from the Genescan^®^ output.

### 2.4 Analysis software

The MLGs were analysed using CLUSTERING CALCULATOR (http://www2.biology.ualberta.ca/jbrzusto/cluster.php), which generated a Phylip DRAWTREE string and bootstrap values (unweighted arithmetic average clustering method, and Jaccard's similarity coefficient), which was converted into a dendrogram by TREEVIEW (http://taxonomy.zoology.gla.ac.uk/rod/treeview.html). Nei's genetic distance (*D*) and Wright's fixation index (*F*_ST_) were calculated using the genetic distance analysis program (GDA; http://lewis.eeb.uconn.edu/lewishome/). The standardised index of association $\left(I_{\text{A}}^{\text{S}}\right)$ was calculated using LIAN 3.1 (Haubold and Hudson, 2000).

## 3 Results

### 3.1 Allele frequencies of new markers

**Fig. 1:**
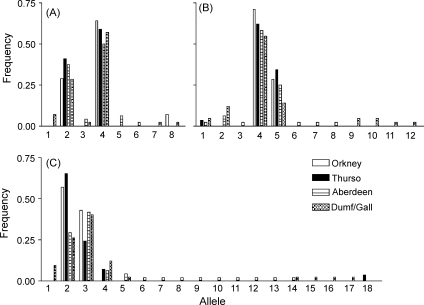
Allele frequencies for microsatellite markers MM5 (graph A), MM18 (B), and MM19 (C), in the four populations studied, Orkney, Thurso, Aberdeenshire and Dumfries and Galloway (Dumf/Gall).

The three new microsatellite markers, MM5, MM18 and MM19, exhibited polymorphism in all *C. parvum* populations in Scotland. The markers had two predominant alleles, with varying numbers of less frequent alleles (Fig. 1). MM19 was the most polymorphic with 18 alleles, followed by MM18 with 12 and MM5 with 8. The additional markers increase the number of loci with at least 2 alleles occurring at frequencies >0.2 from three to six. The four predominantly mono-allelic markers for Scottish samples (Caccio, MS1, MS5 and MS9) are, however, polymorphic for Peruvian samples (data not shown). The new markers did not significantly increase the detection of mixed infections, with these being defined as those with >1 allele at one or more loci. The percentage of mixed infections as detected solely by the three new markers was 10.1% in the Orkney population (8% with the previous 6 markers), 26.8% for Thurso (previously 20%), 22.2% for Dumfriesshire (previously 24%) and 37% for Aberdeenshire (previously 32%).

### 3.2 Multilocus genotypes

**Fig. 2:**
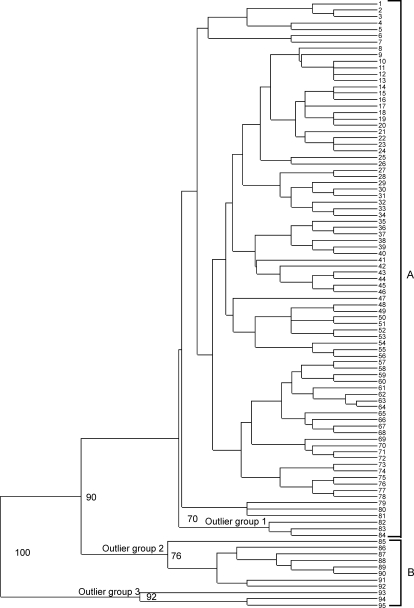
Dendrogram of multilocus genotypes (MLGs) 1-95 as generated by TREEVIEW from CLUSTERING CALCULATOR. Group A consists of samples from humans, cattle and sheep, while Group B consists of samples from humans only. Bootstrap values as calculated by CLUSTERING CALCULATOR are shown at the relevant node; only values greater than 70 are indicated. Outlier groups 1, 2 and 3 are also indicated.

The data from the new microsatellite markers were combined with that published previously (Mallon et al., 2003b). Amplification products were obtained for all samples, with no missing data points over ten markers. 95 distinct MLGs were identified (Supplementary Table 1), a significant increase from the 48 identified previously (Mallon et al., 2003b). Indeed, 10 of the previously identified MLGs were not included in the present analysis due to exhaustion of the samples (MLGs 4, 5, 15, 29, 31, 41, 47, 48, 51 and 52 from Mallon et al. (2003b)), and therefore the increase in MLGs using the new polymorphic markers is actually an expansion from a baseline of the 38 MLGs previously described. To evaluate the similarity of the MLGs, Jaccard's coefficient was calculated and a dendrogram of similarity constructed (Fig. 2). Most of the MLGs cluster in a single large group (A, Fig. 2) comprising samples from human, bovine and ovine hosts from all regions of Scotland (corresponding to sub-group B from Mallon et al. (2003b)). Three more distinct clusters were identified (outlier groups 1, 2 and 3, Fig. 2), with bootstrap values supporting classification as distinct groups. Outlier group 1 consists of one cattle and two human samples and is a novel group defined by the new markers, whereas outlier groups 2 and 3 contain samples only from humans in Aberdeenshire and Dumfriesshire, and correspond to sub-groups A and C described previously (Mallon et al., 2003b). Typing with the additional markers has increased the number of MLGs in outlier groups 2 and 3 from 9 to 14.

### 3.3 Overall population analysis

MLGs were used to analyse the population structure by determining evidence for frequent and random genetic exchange (panmixia). Linkage disequilibrium was measured between alleles at all pairwise combinations of loci using the index of association $\left(I_{\text{A}}^{\text{S}}\right)$ (Haubold and Hudson, 2000), which has a value of zero for panmixia, but a positive value if linkage disequilibrium is detected. Initially samples were pooled as a single population (Table 1A). Analysis was carried out on three levels (for sample sizes see Table 1). Firstly all samples were included (‘All’, Table 1). Secondly, samples from the same postcode with identical MLGs were removed (‘Postcode’ Table 1), negating geographic bias by preventing over-representation of MLGs. Finally, samples with identical MLGs were represented by a single data point (‘MLG’ Table 1). This last layer of analysis allowed the testing of whether the population structure could be epidemic; by removing MLG replicates one can identify masking of underlying panmixia by expansion of particular genetic types (Maynard-Smith et al., 1993). Maynard-Smith described three basic population structures; at one extreme is panmixia, whereby unrestricted mating leads to free genetic exchange, and at the opposite end of the scale is clonality, where there is very limited genetic exchange and relative genetic isolation of any particular genetic type. In between clonal and panmictic are epidemic populations, where rapid expansion of particular genetic types masks underlying genetic exchange. The value of $I_{\text{A}}^{\text{S}}$ was positive at all levels of analysis for the whole Scottish population, indicating linkage disequilibrium (Table 1A). Possible explanations for this result include the presence of more than one genetically isolated population, or a lack of significant genetic exchange. To test the former possibility, the genetically distinct outlier groups 1, 2 and 3 (Fig. 2) were excluded as they represented genetic sub-populations. Analysis carried out after removing these samples indicated that linkage disequilibrium is present when all samples were analysed and also when only one sample/postcode is examined (Table 1A, Scotland^c^). However, the population is in linkage equilibrium when analysis is undertaken with identical MLGs treated as single data points. Therefore, the *C. parvum* population in Scotland has an epidemic population structure. The program used to analyse the data (Haubold and Hudson, 2000) indicates the statistical robustness of the values obtained, by calculating the variance of pairwise differences (*V*_D_) and the 95% critical value of this (*L*), such that if *V*_D_ is not greater than *L*, there is good fit to the null hypothesis of panmixia. Additionally, a probability (*P*) is calculated that the observed pairwise allele frequencies fit a simulation of these frequencies, based on random mating (Table 1).

Analysis was also undertaken on the individual populations (labelled by geographic region, Table 1B). When analysed in isolation, it is evident that Orkney and Thurso are epidemic populations, as there is linkage equilibrium when MLG replicates are removed (Table 1B). In contrast, the Aberdeenshire and Dumfriesshire populations are similar to the population overall, in that linkage disequilibrium is present even when only single MLGs are considered (Table 1B, populations with genetically distinct outliers included). When the Dumfriesshire and Aberdeenshire populations are examined without the genetically distinct outlier populations, they are in linkage equilibrium at all levels of analysis, indicating panmixia. Therefore, the *C. parvum* population structure is panmictic in Dumfriesshire and Aberdeenshire, but epidemic in Orkney and Thurso. The main difference is that the Dumfriesshire and Aberdeenshire sample sets include isolates from human infections. However, if only cattle isolates are analysed, the difference in population structure between Dumfriesshire/Aberdeenshire and Orkney/Thurso is maintained (data not shown). If the human sample data set is analysed in isolation, the results indicate an epidemic structure (data not shown). However, the human data set is small, and caution is attached to this interpretation. In conclusion, the *C. parvum* population in Scotland has an epidemic population structure. However, there are differences at a local geographic level, with Thurso and Orkney exhibiting an epidemic structure, and Aberdeenshire and Dumfriesshire showing evidence of panmixia.

### 3.4 Genetic distance and *F*_ST_ values

Nei's genetic distance (*D*) was used to compare the different populations (Table 2). Outlier groups 1, 2 and 3 were considered as separate populations. The genetic distance between the regional populations was small (all values of *D* < 0.08, Table 2). The most similar were Dumfriesshire and Aberdeenshire, where *D* = 0.0037, compared with values of 0.057 and 0.072 between Dumfriesshire, and Thurso and Orkney, respectively. The difference observed between Orkney and Thurso (*D* = 0.073) was surprisingly the largest measured between the four populations. This is unexpected, as they are the closest geographically, but it must be stressed that *D* is still low. The *F*_ST_ values, a measure of population sub-structure, agree with this trend (Table 2). The parameter *θ* (Hartl and Clark, 1997), an estimate of *F*_ST_, indicated little genetic differentiation between Thurso, Aberdeenshire and Dumfriesshire (*θ* < 0.035 in all cases), but moderate differentiation between Orkney and the other populations. Interestingly, the data combine to suggest that the Orkney *C. parvum* population is more distinct than those in the other regions. The most likely explanation for this is the low level of allele polymorphism found in the Orkney samples, resulting in allele bias, perhaps resulting from the Orkneys being islands with limited movements of cattle.

Each outlier group shows significant genetic difference from the main population samples. The group 1 outliers, comprising 2 bovine (MLGs 82 and 83, Fig. 2) and 1 human (MLG 84) sample, are closer to the main population by clustering analysis (Fig. 2) and genetic distance (*D* = 0.32; Table 3), than either group 2 (*D* = 0.72) or group 3 (*D* = 1.52). However, all three groups are genetically distinct, further evidenced by the *F*_ST_ values (*θ* = 0.126, 0.141 and 0.175 for groups 1, 2 and 3, respectively) indicating a high level of genetic differentiation (Hartl and Clark, 1997). These data suggest there are genetically different subpopulations of *C. parvum* circulating within Scotland. Although the samples in outlier group 1 are from both bovine and human samples, groups 2 and 3 have only been isolated from humans.

### 3.5 Comparison with geographically distinct samples

**Fig. 3:**
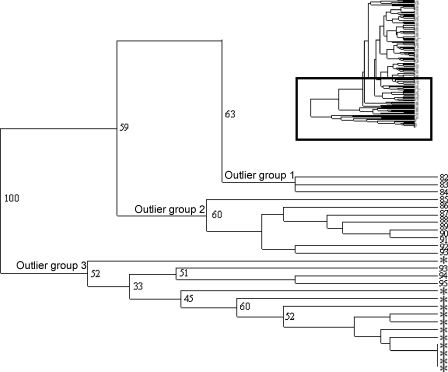
Dendrogram of multilocus genotypes including Peruvian samples. Enlarged section of dendrogram (indicated by dark rectangle on miniature) generated by TREEVIEW from CLUSTERING CALCULATOR. Peruvian isolates are indicated by *. Bootstrap values as calculated by CLUSTERING CALCULATOR are shown for major nodes.

While the analysis of the Scottish samples provides no real evidence of geographic sub-structuring, it was important to test for this with more geographically distinct isolates, particularly as this might provide an explanation for the outlier groups described in Section 3.2. A set of 12 Peruvian *C. parvum* human samples was analysed as a geographically distinct reference set and was genotyped with the 10 markers. Nei's genetic distance and *F*_ST_ were estimated between the main Scottish population, the three outlier groups and the Peruvian samples, and the results indicate that the Peruvian samples are different from all Scottish samples (Table 3). However, when pairwise similarities of the MLGs are presented as a dendrogram (Fig. 3), the Peruvian samples cluster with outlier group 3. In addition, the Peruvian samples are more similar by genetic distance and *F*_ST_ to group 3 (*D* = 0.96, *θ* = 0.49) than to groups 1 and 2 (*D* = 2.15 and 2.11, *θ* = 0.633 and 0.613, respectively) or the main Scottish *C. parvum* population (*D* = 2.39, *θ* = 0.50).

### 3.6 Correlation of allele polymorphism with cattle movements

**Fig. 4:**
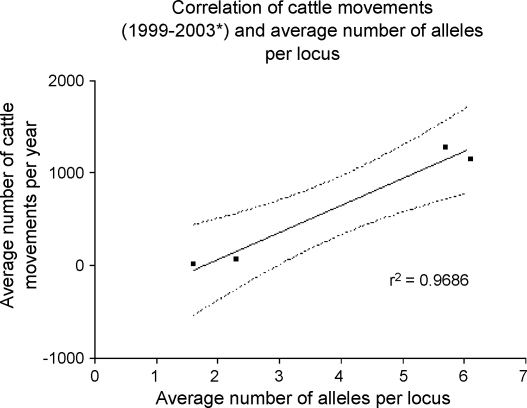
Correlation of cattle movements and average number of alleles per locus. Cattle data from British Cattle Movement Service, DEFRA. Data were for cattle killed (on farm) in the study area that were born outside of any of the four study areas (*data for 2001 not included due to FMD outbreak). Shown are the goodness of fit value (*r*^2^), and 95% confidence intervals (dashed lines).

Cattle movements in the four regions were obtained from the British Cattle Movement Service (DEFRA; www.bcms.gov.uk) and provided data on the number of cattle killed (on farm) in each area that were born outside of the respective study areas, for the years 1999–2003. Data for 2001 were not included due to the FMD outbreak. When this measure of host movement was plotted against the average number of *C. parvum* alleles per locus from each geographic area (Fig. 4), a strong association was observed (correlation coefficient *r*^2^ = 0.9686). Therefore, the data suggest that the movement of cattle into a region contributes to the genetic diversity of *C. parvum*.

## 4 Discussion

### 4.1 Genetic exchange and geographical sub-structuring

The key question addressed by the data presented is whether the conclusions of two previous analyses (Mallon et al., 2003a,b) of the population genetics of *C. parvum* in Scotland are confirmed by the use of additional polymorphic microsatellite markers. In the previous studies the populations from Orkney, Thurso and Aberdeen were found to be panmictic. The new data show that the Orkney and Thurso populations are in linkage disequilibrium unless replicate MLGs are treated as a single data point. Therefore, these populations have an epidemic population structure with certain MLGs over-represented and masking underlying mating. The different conclusion between this and the previous study is due to the inclusion of three additional polymorphic markers, giving the analysis higher resolution by testing appreciable predicted frequencies of pairwise allele combinations against the null hypothesis of panmixia. Thus, previously a type 2 error resulted from the near monomorphy of some markers, as the frequency of allele combinations with random genetic exchange would not be significantly different from those if mating was not occurring. For the Aberdeen population, excluding the distinct outlier subgroups, the new analysis confirms the previous conclusion of panmixia and frequent mating. The re-analysis of the Dumfriesshire population combining both human and bovine samples, but excluding the isolates from the outlier groups, showed that this was also panmictic. In both Aberdeenshire and Dumfriesshire, the inclusion of the distinct human outlier groups in the analysis led to linkage disequilibrium providing evidence for genetic sub-structuring in these populations. It must be stressed that all of the samples included in our analysis are *C. parvum* by COWP RFLP, and therefore the differences we are describing are not comparable to the classically described different ‘host genotypes’ in the *Cryptosporidium* literature (Xiao et al., 2004), but are evidence for significant genetic diversity within *C. parvum sensu stricto*.

The previous studies did not examine the population structure of the Scottish samples treated as a single population. However, Nei's genetic distance and the inbreeding coefficient *F*_ST_ were measured and, based on the low values, it was concluded that there was little geographical sub-structuring across Scotland. This conclusion is supported by the present analysis, excluding the three outlier groups, as a single population that shows an epidemic population structure (although it should be pointed out that longitudinal analysis would be required to confirm if this scenario is stable over time). The over-representation of specific MLGs results from the inclusion of the Thurso and Orkney populations and implies greater gene flow within and between Aberdeenshire and Dumfriesshire. This is supported by the genetic distance and *F*_ST_ between populations, which show limited differentiation and low values between Aberdeenshire and Dumfriesshire. Thus, although there is random genetic exchange occurring across the whole population, the two northern-most populations demonstrate an epidemic structure with certain MLGs predominating. The data also provide evidence against any significant host or geographical sub-structuring.

### 4.2 Host movement and population diversity

The positive correlation between parasite genetic diversity and cattle movements indicates an important role for the bovine host in the spread and generation of parasite genetic diversity. The prevalence of human *C. parvum* infections in Europe, compared with the predominance of *C. hominis* in other regions, has led to the suggestion that intensive agriculture in European countries is linked to the higher prevalence of *C. parvum* human outbreaks (Xiao and Ryan, 2004). Our data suggest that the *C. parvum* population circulates and spreads via cattle in Scotland. A similar finding regarding the influence of cattle movements on parasite populations has been described. A greater number of genetic types per farm was found in Turkey, where cattle mix regularly, whereas in Israel cattle movements are more restricted, leading to lower genetic diversity (Tanriverdi et al., 2006). The findings of different geographic population structures and the correlation of allelic diversity with cattle movements suggest a heavy influence of host density and movements on the parasite population. This therefore provides potential for the parasite population structure in different regions or countries to be determined by host population movements and, in the case of cattle, the structure of the livestock industry, with implications for epidemiology and control measures. However, as with many pathogen population studies, there is an inherent bias in the present study that samples are from symptomatic cases, and to fully explore the population genetics of *C. parvum*, sampling of both symptomatic and asymptomatic hosts would be needed.

### 4.3 Genetic sub-structuring

A small subset of human samples from Aberdeen and Dumfriesshire, genetically distinct from the main population, had previously been reported (Mallon et al., 2003a,b) and these results are confirmed. From population genetic analysis, these MLGs are genetically isolated from the main Scottish *C. parvum* population and measures of both genetic distance and *F*_ST_ show that they are substantially different from each other and the rest of the population. The genetic distances (*D*) are comparable to those between the related species of the *Drosophila melanogaster* complex (Harr et al., 1998). The identification of these distinct groups raises questions about their origins and status in terms of being members of the same species. These samples comprise a small proportion of the *C. parvum* samples analysed (4.8%), indicating that they are a rare source of infection, or rarely cause disease. Whether this is because they are from an unidentified source of infection, or there is (for groups 2 and 3) a human–human transmission cycle, requires further study. The grouping of Peruvian *C. parvum* human samples with outlier group 3 raises the possibility that the minor group of samples represented by the outlier groups are brought into circulation either by animal import or human travel, and, at the time of sampling, had not undergone recombination with the indigenous Scottish population, explaining their significant genetic difference. Indeed, of the MLGs in outlier group 3, two (MLGs 94 and 95, Fig. 2) were cases from humans who had travelled abroad, although the destination was not recorded. In order to follow up this interpretation, comparison with a comprehensive panel of geographically diverse samples would be required. These data provide an indication that the genetically distinct isolates are being imported by host movements, further emphasising the role of hosts in increasing parasite genetic diversity. To fully understand the genetic diversity of the *C. parvum* parasite, a large sample set from geographically diverse regions needs to be compared, in order to examine the degree of genetic differentiation and potential gene flow between regions. Whether the outlier samples can interbreed with the main population is uncertain as they have possibly been recently imported and thus not extensively transmitted. However, if the genetic differences between Peruvian and Scottish *C. parvum* are a feature of geographically well-separated populations, then it could be possible to identify the origin of imported strains. Longitudinal studies would be required to establish if these genetically distinct parasites could undergo genetic exchange with the local population.

## Figures and Tables

**Table 1:** Linkage equilibrium and disequilibrium in Scottish *C. parvum* populations

Population	Analysis^a^	*n*	Standardised *I*_A_^b^	*P*-value	*V*_D_ > *L*	LE or LD
A						
Scotland	All	297	0.1373	0.01	Y	LD
	Postcode	199	0.1725	0.001	Y	LD
	MLG	132	0.1436	0.01	Y	LD

Scotland^c^	All	275	0.0313	0.01	Y	LD
	Postcode	178	0.0058	0.05	Y	LD
	MLG	110	−0.008	0.97	N	LE

B						
Orkney	All	68	0.0229	0.001	Y	LD
	Postcode	30	0.0995	0.001	Y	LD
	MLG	14	−0.0204	0.882	N	LE

Thurso	All	96	0.0322	0.001	Y	LD
	Postcode	46	0.0267	0.006	Y	LD
	MLG	28	0.0005	0.479	N	LE

Aberdeen	All	70	0.1368	0.01	Y	LD
	Postcode	65	0.1391	0.01	Y	LD
	MLG	48	0.1282	0.01	Y	LD

Aberdeen^c^	All	60	0.0087	0.159	N	LE
	Postcode	55	0.0084	0.141	N	LE
	MLG	38	−0.0137	0.91	N	LE

Dumf/Gall	All	63	0.3869	0.001	Y	LD
	Postcode	58	0.3968	0.01	Y	LD
	MLG	42	0.3014	0.001	Y	LD

Dumf/Gall^c^	All	50	0.0105	0.172	N	LE
	Postcode	47	0.0088	0.196	N	LE
	MLG	32	−0.0094	0.788	N	LE

a Level of analysis.

b *Abbreviations*: standardised *I*_A_, standardised index of association; *V*_D_, variance of pairwise differences; *L*, 95% critical value for *V*_D_; LE, linkage equilibrium; LD, linkage disequilibrium; A, all samples; B, individual populations; MLG, multilocus genotype.

c Populations with genetically distinct outliers removed.

**Table 2:** Genetic distance (*D*) and *F*_ST_ (*θ*) values between Orkney, Thurso, Aberdeenshire and Dumfries and Galloway (Dumf/Gall)

Measurement^a^	Orkney	Thurso	Aberdeenshire	Dumf/Gall
*D* (Orkney)	–			
*D* (Thurso)	0.073153	–		
*D* (Aberdeenshire)	0.064992	0.032677	–	
*D* (Dumf/Gall)	0.072421	0.057286	0.003768	–

*θ*	0.069743	0.030613	0.006433	0.034208

a Genetically distinct Outlier Group samples removed from data set.

**Table 3:** Genetic distance (*D*) and *F*_ST_ (*θ*) values between samples from Scotland, Outlier Groups 1, 2 and 3 and Peruvian samples

Measurement	Scotland	Outliers 1	Outliers 2	Outliers 3	Peru
*D* (Scotland)	–	0.32831	0.71649	1.52109	2.39709
*θ* (Scotland)	–	0.126780	0.141847	0.175824	0.49689

*D* (Peru)	2.39709	2.14968	2.11402	0.9597	–
*θ* (Peru)	0.49689	0.633505	0.612830	0.495611	–
