# Supplementary material for: The population structure of the Cryptosporidium parvum population in Scotland: A complex picture
Source: Infect Genet Evol. 2008 Mar;8(2-3):121–9. doi: 10.1016/j.meegid.2007.10.010 (PMC2684618; doi:10.1016/j.meegid.2007.10.010)
Supplement: Supplementary file 1 [file mmc1.doc]

**APPENDIX**

Supplementary Table 1. Multilocus genotypes (MLGs) identified. Each MLG is assigned a number (1 – 95). The designated allele for each marker (MS1, Caccio, MS5, TP14, MS9, GP15, MS12, MM5, MM18 and MM19) is shown for each MLG, and the corresponding MLG from (Mallon et al 2003b) is displayed (Mallon MLG).

| **MLG** | **MS1** | **MS9** | **TP14** | **MS5** | **GP15** | **ML1** | **MS12** | **MM5** | **MM18** | **MM19** | **Mallon MLG** |
| --- | --- | --- | --- | --- | --- | --- | --- | --- | --- | --- | --- |
| **1** | 3 | 5 | 5 | 9 | 9 | 5 | 1 | 4 | 5 | 4 | 12 |
| **2** | 3 | 5 | 5 | 9 | 6 | 5 | 1 | 4 | 5 | 4 | 9 |
| **3** | 3 | 5 | 5 | 9 | 6 | 5 | 2 | 4 | 5 | 4 | 8 |
| **4** | 3 | 10 | 5 | 9 | 5 | 5 | 1 | 4 | 4 | 4 | 44 |
| **5** | 3 | 10 | 5 | 9 | 5 | 5 | 2 | 4 | 5 | 4 | 45 |
| **6** | 3 | 7 | 5 | 9 | 6 | 5 | 2 | 2 | 4 | 4 | 50 |
| **7** | 3 | 7 | 5 | 9 | 5 | 5 | 1 | 2 | 4 | 4 | 16 |
| **8** | 3 | 5 | 5 | 6 | 6 | 5 | 2 | 2 | 4 | 3 | 14 |
| **9** | 3 | 5 | 5 | 9 | 6 | 5 | 2 | 2 | 1 | 3 | 8 |
| **10** | 3 | 5 | 5 | 9 | 7 | 5 | 2 | 2 | 4 | 3 | 10 |
| **11** | 3 | 5 | 5 | 9 | 5 | 5 | 2 | 2 | 4 | 3 | 6 |
| **12** | 3 | 5 | 5 | 9 | 8 | 5 | 2 | 2 | 4 | 3 | 11 |
| **13** | 3 | 5 | 5 | 9 | 6 | 5 | 2 | 2 | 4 | 3 | 8 |
| **14** | 3 | 5 | 5 | 9 | 5 | 5 | 2 | 4 | 4 | 3 | 6 |
| **15** | 3 | 5 | 5 | 9 | 5 | 5 | 2 | 3 | 10 | 3 | 6 |
| **16** | 3 | 5 | 5 | 9 | 5 | 5 | 2 | 4 | 5 | 3 | 6 |
| **17** | 3 | 5 | 5 | 9 | 7 | 5 | 2 | 4 | 5 | 3 | 10 |
| **18** | 3 | 5 | 5 | 9 | 9 | 5 | 2 | 4 | 4 | 3 | 40 |
| **19** | 3 | 5 | 5 | 9 | 7 | 5 | 2 | 3 | 4 | 3 | 10 |
| **20** | 3 | 5 | 5 | 9 | 18 | 5 | 2 | 4 | 4 | 3 | 54 |
| **21** | 3 | 5 | 5 | 9 | 6 | 5 | 2 | 5 | 4 | 3 | 8 |
| **22** | 3 | 5 | 5 | 9 | 6 | 5 | 2 | 4 | 4 | 3 | 8 |
| **23** | 3 | 5 | 5 | 9 | 6 | 5 | 2 | 4 | 9 | 3 | 8 |
| **24** | 3 | 5 | 5 | 9 | 6 | 5 | 2 | 4 | 5 | 3 | 8 |
| **25** | 3 | 5 | 5 | 9 | 8 | 5 | 2 | 3 | 4 | 4 | 11 |
| **26** | 3 | 5 | 5 | 9 | 7 | 5 | 2 | 4 | 4 | 14 | 10 |
| **27** | 3 | 5 | 5 | 9 | 5 | 5 | 2 | 2 | 5 | 4 | 6 |
| **28** | 3 | 5 | 5 | 9 | 5 | 5 | 2 | 2 | 5 | 2 | 6 |
| **29** | 3 | 5 | 5 | 9 | 6 | 5 | 2 | 2 | 4 | 11 | 8 |
| **30** | 3 | 5 | 5 | 9 | 6 | 5 | 2 | 2 | 4 | 2 | 8 |
| **31** | 3 | 5 | 5 | 9 | 6 | 5 | 2 | 2 | 5 | 2 | 8 |
| **32** | 3 | 5 | 5 | 9 | 8 | 5 | 2 | 2 | 4 | 2 | 11 |
| **33** | 3 | 5 | 6 | 9 | 5 | 5 | 2 | 2 | 4 | 2 | 22 |
| **34** | 3 | 5 | 5 | 9 | 5 | 5 | 2 | 2 | 4 | 2 | 6 |
| **35** | 3 | 5 | 5 | 9 | 6 | 5 | 1 | 4 | 4 | 8 | 9 |
| **36** | 3 | 5 | 5 | 9 | 6 | 5 | 2 | 4 | 4 | 2 | 8 |
| **37** | 3 | 5 | 5 | 9 | 6 | 5 | 1 | 4 | 4 | 2 | 9 |
| **38** | 3 | 5 | 5 | 9 | 6 | 5 | 1 | 2 | 4 | 3 | 9 |
| **39** | 3 | 5 | 5 | 9 | 6 | 5 | 1 | 4 | 4 | 3 | 9 |
| **40** | 3 | 5 | 5 | 9 | 5 | 5 | 1 | 4 | 4 | 3 | 7 |
| **41** | 3 | 5 | 5 | 9 | 6 | 5 | 1 | 2 | 2 | 2 | 9 |
| **42** | 3 | 5 | 6 | 9 | 5 | 5 | 1 | 2 | 4 | 14 | 23 |
| **43** | 3 | 5 | 5 | 9 | 20 | 5 | 1 | 2 | 4 | 2 | 56 |
| **44** | 3 | 5 | 5 | 9 | 5 | 5 | 1 | 2 | 4 | 2 | 7 |
| **45** | 3 | 5 | 5 | 9 | 5 | 5 | 1 | 2 | 4 | 18 | 7 |
| **46** | 3 | 5 | 5 | 9 | 5 | 5 | 1 | 2 | 4 | 3 | 7 |
| **47** | 2 | 5 | 5 | 9 | 19 | 5 | 1 | 4 | 4 | 2 | 58 |
| **48** | 3 | 6 | 5 | 9 | 5 | 5 | 1 | 4 | 4 | 2 | 46 |
| **49** | 3 | 5 | 5 | 9 | 5 | 5 | 1 | 4 | 4 | 2 | 7 |
| **50** | 3 | 5 | 5 | 9 | 5 | 5 | 1 | 4 | 5 | 2 | 7 |
| **51** | 3 | 5 | 5 | 9 | 5 | 5 | 2 | 4 | 5 | 2 | 6 |
| **52** | 3 | 5 | 5 | 11 | 5 | 5 | 2 | 4 | 4 | 2 | 13 |
| **53** | 3 | 5 | 5 | 9 | 5 | 5 | 2 | 4 | 4 | 2 | 6 |
| **54** | 5 | 5 | 5 | 9 | 5 | 5 | 2 | 4 | 4 | 3 | 30 |
| **55** | 5 | 5 | 5 | 9 | 5 | 5 | 2 | 3 | 4 | 2 | 30 |
| **56** | 5 | 5 | 5 | 9 | 5 | 5 | 2 | 4 | 4 | 2 | 30 |
| **57** | 3 | 5 | 6 | 9 | 6 | 5 | 2 | 4 | 4 | 15 | 24 |
| **58** | 3 | 5 | 6 | 9 | 6 | 5 | 2 | 4 | 4 | 2 | 24 |
| **59** | 3 | 5 | 6 | 9 | 6 | 5 | 2 | 2 | 4 | 3 | 24 |
| **60** | 3 | 5 | 6 | 9 | 6 | 5 | 2 | 2 | 4 | 2 | 24 |
| **61** | 3 | 5 | 6 | 9 | 5 | 5 | 2 | 8 | 4 | 3 | 22 |
| **62** | 3 | 5 | 6 | 9 | 6 | 5 | 2 | 4 | 4 | 3 | 24 |
| **63** | 3 | 5 | 6 | 9 | 6 | 5 | 2 | 4 | 4 | 3 | 24 |
| **64** | 3 | 5 | 6 | 9 | 5 | 5 | 2 | 4 | 4 | 3 | 22 |
| **65** | 3 | 6 | 6 | 9 | 5 | 5 | 2 | 4 | 4 | 2 | 26 |
| **66** | 3 | 5 | 6 | 9 | 7 | 5 | 2 | 4 | 4 | 2 | 42 |
| **67** | 3 | 5 | 6 | 9 | 5 | 5 | 2 | 4 | 4 | 4 | 22 |
| **68** | 3 | 5 | 6 | 9 | 5 | 5 | 2 | 4 | 4 | 2 | 22 |
| **69** | 3 | 5 | 6 | 9 | 5 | 5 | 1 | 4 | 5 | 3 | 23 |
| **70** | 3 | 6 | 6 | 9 | 5 | 5 | 2 | 4 | 5 | 3 | 26 |
| **71** | 3 | 5 | 6 | 9 | 5 | 5 | 2 | 4 | 5 | 3 | 22 |
| **72** | 3 | 5 | 6 | 9 | 5 | 5 | 2 | 4 | 5 | 2 | 22 |
| **73** | 3 | 5 | 6 | 9 | 6 | 5 | 1 | 4 | 10 | 2 | 25 |
| **74** | 3 | 5 | 6 | 9 | 6 | 5 | 1 | 4 | 5 | 2 | 25 |
| **75** | 3 | 5 | 5 | 9 | 6 | 5 | 2 | 4 | 5 | 2 | 8 |
| **76** | 3 | 5 | 6 | 9 | 6 | 5 | 2 | 4 | 5 | 2 | 24 |
| **77** | 3 | 5 | 6 | 9 | 6 | 5 | 2 | 8 | 5 | 2 | 24 |
| **78** | 3 | 5 | 6 | 9 | 6 | 5 | 2 | 2 | 5 | 2 | 24 |
| **79** | 3 | 5 | 5 | 9 | 5 | 5 | 1 | 6 | 5 | 3 | 7 |
| **80** | 7 | 5 | 5 | 9 | 5 | 5 | 1 | 2 | 5 | 2 | 43 |
| **81** | 4 | 5 | 5 | 9 | 5 | 5 | 1 | 4 | 5 | 9 | 28 |
| **82** | 3 | 6 | 5 | 9 | 19 | 5 | 2 | 2 | 9 | 3 | 55 |
| **83** | 3 | 6 | 5 | 9 | 6 | 5 | 2 | 4 | 2 | 3 | 27 |
| **84** | 3 | 6 | 5 | 9 | 6 | 5 | 2 | 2 | 3 | 3 | 27 |
| **85** | 3 | 4 | 6 | 7 | 6 | 2 | 2 | 3 | 4 | 6 | 17 |
| **86** | 3 | 4 | 6 | 9 | 8 | 3 | 2 | 2 | 2 | 13 | 21 |
| **87** | 3 | 4 | 6 | 9 | 2 | 2 | 2 | 2 | 2 | 12 | 18 |
| **88** | 3 | 4 | 6 | 9 | 17 | 2 | 2 | 3 | 2 | 5 | 53 |
| **89** | 3 | 4 | 6 | 9 | 17 | 2 | 2 | 2 | 2 | 17 | 53 |
| **90** | 3 | 4 | 6 | 9 | 17 | 2 | 2 | 2 | 2 | 16 | 53 |
| **91** | 3 | 4 | 6 | 9 | 6 | 2 | 2 | 5 | 8 | 10 | 20 |
| **92** | 3 | 4 | 6 | 9 | 3 | 2 | 2 | 5 | 2 | 7 | 19 |
| **93** | 1 | 6 | 4 | 2 | 16 | 3 | 5 | 2 | 12 | 2 | 57 |
| **94** | 1 | 8 | 4 | 4 | 5 | 3 | 2 | 2 | 7 | 5 | 2 |
| **95** | 1 | 9 | 4 | 2 | 5 | 3 | 2 | 2 | 6 | 5 | 3 |
